# Análisis comparativo según la clasificación de la Organización Mundial de la Salud 2005 y 2017 frente a la OMS 2022 en tumores de glándulas salivales

**DOI:** 10.21142/2523-2754-1403-2026-302

**Published:** 2026-07-10

**Authors:** Karina Gabriela Moo-Pool, Javier Portilla-Robertson, José de Jesús Ramos-Nieto, Claudia Patricia Mejía-Velázquez, Luis Fernando Jacinto-Alemán

**Affiliations:** 1 División de Estudios de Posgrado e Investigación, Facultad de Odontología UNAM. Ciudad de México, México. jpr@unam.mx, dramejiavelazquez@fo.odonto.unam.mx , Ifja@fo.odonto.unam.mx Universidad Nacional Autónoma de México División de Estudios de Posgrado e Investigación Facultad de Odontología UNAM Ciudad de México Mexico jpr@unam.mx dramejiavelazquez@fo.odonto.unam.mx Ifja@fo.odonto.unam.mx; 2 Facultad de Odontología UNAM. Ciudad de México, México. venison.13@hotmail.com Universidad Nacional Autónoma de México Facultad de Odontología UNAM Ciudad de México Mexico venison.13@hotmail.com

**Keywords:** tumores de glándula salival, adenoma pleomorfo, cáncer de glándulas salivales, carcinoma mucoepidermoide, salivary gland tumors, cancer of salivary glands, pleomorphic adenoma, mucoepidermoid carcinoma

## Abstract

**Objetivo.:**

Las neoplasias de glándula salival constituyen un grupo heterogéneo. Por esta variabilidad, las clasificaciones de la OMS (Organización Mundial de la Salud) se actualizan para incorporar los nuevos hallazgos. El objetivo del presente estudio es analizar los diagnósticos histopatológicos de tumores de glándula salival clasificados bajo las clasificaciones de 2005 y 2017, y contrastarlos con la de 2022, para describir las características demográficas de los tumores de glándula salival.

**Metodología.:**

Se realizó un estudio retrospectivo y descriptivo, en el que revisamos un total de 13,067 casos, de enero de 2005 a diciembre de 2023. Se seleccionaron 115 casos de tumores de glándulas salivales, pero se incluyeron 97 en el análisis. La información demográfica, como edad y sexo, se obtuvo de los reportes histopatológicos. La reclasificación histológica se realizó mediante el análisis microscópico del tejido, teñido en H&E (tinción de hematoxilina y eosina).

**Resultados.:**

Se observó que, en la clasificación OMS 2005, los papilomas constituyeron el 2% de la muestra, mientras que en la de 2022 representaron el 1%, al considerarse al sialadenoma papilliferum como entidad en sí misma desde 2017. En cuanto a las neoplasias malignas, se observó que, en la clasificación OMS 2017, el término adenocarcinoma NOS (no especificado de otra manera) representó el 2%, mientras que en la de 2022 el término se sustituye por el de carcinoma salival NOS y entidades emergentes, que representaron el 2% de la muestra.

**Conclusión.:**

Los cambios en la nomenclatura de la OMS para los tumores de glándulas salivales pueden sugerir variaciones en la frecuencia de los datos. Sin embargo, limitantes como la falta de herramientas diagnósticas, no nos permiten establecer con mayor claridad los diagnósticos y, de esta manera, brindar datos más certeros.

## Abstract (en)

**Objective.:**

Salivary gland neoplasms constitute a heterogeneous group. Due to this variability, the WHO (World Health Organization) classifications are updated to incorporate the new findings. The objective of this study is to reclassify the histopathological diagnoses of salivary gland tumors classified under the WHO 2005 and 2017 and contrasting with the WHO 2022, describing the demographic characteristics of salivary gland tumors.

**Methodology.:**

A retrospective and descriptive study was reviewed, in which we reviewed total of 13067 cases, from January 2005 to December 2023, 115 cases of salivary gland tumors were selected, but 97 were included in the analysis. Demographic information, such as age and sex, was obtained from histopathological reports. The histological reclassification was carried out through the microscopic analysis of the tissue, stained in H&E (hematoxylin and eosin stain).

**Results.:**

It was observed for the WHO 2005 the papilloma constituted 2% of the sample being that by 2022 they represented 1% when considering the sialoadenoma papilliferum as entity since 2017; as for malignant neoplasms it was observed that for the WHO 2017 the term adenocarcinoma NOS (Not Otherwise Specified) represented 2%; for the WHO 2022 the term is replaced by that of Salivary Carcinoma NOS and Emerging entities representing 2% of the sample.

**Conclusion.:**

Changes in the WHO nomenclature for salivary gland tumors may suggest variations in the frequency of these findings. However, limitations such as the lack of diagnostic tools would help us more clearly establish diagnoses and thus provide more accurate data.

## INTRODUCCIÓN

Las neoplasias de glándulas salivales representan un desafío diagnóstico para los especialistas en patología debido a la complejidad de sus características morfológicas y la superposición de patrones histológicos, lo que resulta en tumores diversos y difíciles de diagnosticar ^1^^-^^4^. Por esta razón, expertos en el campo han unido esfuerzos para desarrollar una guía que ayude a consolidar las características de las neoplasias de las glándulas salivales ^4^^,^^5^.

La Organización Mundial de la Salud (OMS) ha sido fundamental a nivel internacional en el desarrollo de un consenso que sirva como guía para el diagnóstico histológico de las neoplasias de las glándulas salivales. Esto ha sido posible gracias a la publicación de su *Libro Azul* sobre tumores de cabeza y cuello ^6^^-^^10^.

Con los avances en la ciencia y la implementación de nuevas herramientas diagnósticas, se han aclarado las incertidumbres anteriores ^4^^,^^11^^,^^12^, y han surgido nuevos hallazgos que requieren cambios en la nomenclatura de las clasificaciones previamente establecidas por la OMS. En consecuencia, esto ha generado confusión respecto a términos previamente utilizados y puede afectar a los datos epidemiológicos sobre neoplasias de glándulas salivales, debido a la falta de registros actualizados alineados con las nuevas categorías que rigen estas patologías en cada clasificación ^1^^,^^6^^,^^13^.

El objetivo del presente estudio fue analizar los diagnósticos histopatológicos de tumores de glándula salival clasificados bajo la OMS 2005 y 2017, y contrastarlos con la OMS 2022, para describir las características demográficas de los tumores de glándula salival.

## MATERIALES Y MÉTODOS

Se realizó un estudio retrospectivo y descriptivo en el que se revisaron un total de 13 067 casos correspondientes al total de biopsias recibidas (sean o no neoplasias de glándula salival) del archivo del Laboratorio de Patología Oral del DEPeI (División de Estudios de Posgrado e Investigación), de la Facultad de Odontología de la UNAM, desde enero de 2005 hasta diciembre de 2023. Mediante un muestreo por conveniencia, se seleccionaron 115 casos de tumores de glándulas salivales, pero se incluyeron 97 en el análisis tras aplicar los criterios de inclusión. Estos criterios consideraron todas las secciones histopatológicas teñidas con H&E y con diagnóstico previo de neoplasia de glándulas salivales benignas o malignas, registradas durante el periodo mencionado.

Los criterios de exclusión incluyeron todas las secciones histopatológicas teñidas con H&E y con diagnóstico previo de neoplasia benigna o maligna de glándulas salivales que, tras un análisis microscópico, no coincidían con las características histológicas de las neoplasias de glándulas salivales; deslizamientos de tejido en mal estado o sin bloques tisulares identificables durante la revisión; y casos en los que el diagnóstico no pudo categorizarse histológicamente según la clasificación de la OMS 2022, dentro de los criterios esenciales histológicos para poder designar una neoplasia de glándula salival, propuestos en la misma clasificación del *Libro azul* (se incluye como anexo una tabla de rúbricas histológicas que se tomaron como base para la revisión de los casos).

La información demográfica, como la edad y el sexo, se obtuvo de informes histopatológicos y formó parte de las variables politómicas nominales. La reclasificación histológica se realizó mediante análisis microscópico de tejido teñido con H&E.

El proyecto cumple con la normativa institucional y los acuerdos de confidencialidad utilizados por la Facultad de Odontología de la Universidad Nacional Autónoma de México (UNAM), implementados en sus clínicas de admisión, en consultas de posgrado como la Clínica de Patología y Medicina Oral, y en el Departamento de Patología Oral y Maxilofacial del DEPeI FO UNAM. Dado que el proyecto se basa en la revisión de diapositivas teñidas con H&E, la identidad de las personas de las que provienen los tejidos no se verá comprometida, ya que no se requiere información personal identificable.

Además, el estudio fue aprobado por el Comité de Ética de la Facultad de Odontología de la UNAM, bajo el número de protocolo asignado: CIE/1111/11/2023.

## RESULTADOS

En cuanto a las características demográficas, se reportaron las distribuciones de frecuencias. Para la variable sexo, las mujeres fueron el grupo más frecuente, pues representaron el 60% (58 casos), mientras que los hombres representaron el 40% (39 casos) (Tabla 1).

Para la variable localización anatómica, el paladar fue el más común, representando el 47% (46 casos), seguido de la región parotídea con el 21% (20 casos). La tercera localización más frecuente fue la mucosa labial, que representó el 11% (11 casos) (Tabla 1).

Se realizó un análisis descriptivo de la edad de los participantes, incluyendo a 95 individuos, ya que en 2 casos no se disponía de este dato. La edad osciló entre un mínimo de 13 años y un máximo de 81 años. La edad media fue de 47.32 años, lo que indica una tendencia central moderada en la muestra. La desviación estándar fue de 16.142 años, lo que sugiere una variabilidad considerable en la edad de los participantes. Al agrupar la edad por décadas, la mayor frecuencia se observó en la cuarta a quinta década de la vida, con el 24% (23 casos); seguida de la quinta a sexta década, con el 18% (17 casos); y en tercer lugar la sexta a séptima década, con el 16% (16 casos) (Tabla 1).

**Tabla 1:** Distribución de sexo, sitio anatómico y edad para tumores de glándula salival (DEPeI, 2005-2023)

Sexo	Frecuencia	Sitio anatómico			Frecuencia		
Hombres 39	(40%)	Paladar			46 (47%)		
Mujeres 58	(60%)	Región parotídea			20 (2%)		
Total 97	100%	Mucosa labial			11 (11%)		
Edad en décadas	Frecuencia	Mucosa yugal			6 (6%)		
11-20	4 (4%)	Región submandibular			5 (5%)		
21-30	13 (13%)	Área retromolar			4 (4%)		
31-40	15 (15%)	Región nasogeniana			1 (1%)		
41-50	23 (24%)	Piso de boca			1 (1%)		
51-60	17 (18%)	No especificado			2 (2%)		
61-70	16 (16%)						
71-80	6 (6%)						
81-90	1 (1%)		N	Mínimo	Máximo	Media	DE
Ne.	2 (2%)	Edad	95	13	81	47.32	16.14

**Tabla 2:** Distribución de frecuencias de tumores de glándulas salivales según las clasificaciones de la OMS (2005, 2017, 2022)

Tumores benignos y malignos de acuerdo con la OMS 2005, 2017 y 2022			
Tumor	Frecuencia 2005	Frecuencia 2017	Frecuencia 2022
Adenoma pleomorfo	58 (60%)	58 (60%)	58 (60%)
Mioepitelioma	9 (9%)	9 (9%)	9 (9%)
Adenoma de células basales	5 (5%)	5 (5%)	5 (5%)
Carcinoma mucoepidermoide	9 (9%)	9 (9%)	9 (9%)
Tumor de Whartin	3 (3%)	3 (3%)	3 (3%)
Papilomas	2 (2%)	1 (1%)	1 (1%)
Cistadenoma	2 (2%)	2 (2%)	2 (2%)
Carcinoma adenoideo quístico	2 (2%)	2 (2%)	2 (2%)
Adenoma canalicular	1 (1%)	1 (1%)	1 (1%)
Adenocarcinoma polimorfo de bajo grado	1 (1%)	1 (1%)	1 (1%)
Carcinoma mioepitelial-epitelial	1 (1%)	1 (1%)	1 (1%)
Adenocarcinoma de células basales	1 (1%)	1 (1%)	1 (1%)
Sialadenoma papilliferum	0 (0%)	1 (1%)	1 (1%)
Cistadenocarcinoma	1 (1%)	0 (0%)	0 (0%)
Adenocarcinoma NOS (No especificado)	1 (1%)	2 (2%)	0 (0%)
Carcinoma ex- adenoma pleomorfo	1 (1%)	1 (1%)	1 (1%)
Carcinoma saliva NOS y entidades emergentes	0 (0%)	0 (0%)	2 (2%)
Total	97 (100%)	97 (100%)	97 (100%)

En cuanto a la distribución en frecuencia de los tumores de glándulas salivales en las tres clasificaciones de la OMS, se identificó el adenoma pleomórfico como el tumor más común en las tres ediciones, con el 60% (58 casos), seguido por el mioepitelioma, con un 9% (9 casos). Entre los tumores malignos, el carcinoma mucoepidermoide fue el más frecuente, ya que representó el 9% (9 casos), seguido por el carcinoma quístico adenoide, con el 2% (2 casos) de la muestra total.

Para los tumores benignos, en la OMS 2005, los papilomas representaban el 2% de la muestra, mientras que en 2022 representaban el 1%, ya que sialadenoma papilliferum se reconoce como una entidad distinta desde 2017. En cuanto a las neoplasias malignas, en la OMS 2017, el término adenocarcinoma NOS representó el 2% (2 casos); en la OMS 2022, este término fue reemplazado por carcinoma salival NOS y entidades emergentes, que también representaba el 2% (2 casos) de la muestra (Tabla 2).

## DISCUSIÓN

Este estudio tuvo como objetivo contrastar los cambios en la nomenclatura de las clasificaciones de la OMS con la edición actual de la OMS de 2022, para describir las características demográficas de los tumores de las glándulas salivales.

Entre los cambios identificados en nuestro estudio, al contrastar la nomenclatura de la OMS de ediciones previas con la clasificación vigente de la OMS 2022, se observó que el 85% (82 casos) de nuestra muestra permanecieron sin modificaciones.

En cuanto a los casos que presentaron cambios, se identificó que el 13% (13 casos) mostró una modificación en la nomenclatura, resultado comparable al reportado por Van der Wal et al. ^14^, quienes encontraron cambios en el 11,7% de su muestra (n = 478) al comparar distintas ediciones de la clasificación de la OMS.

De los casos con cambio de nomenclatura, el 11% (11 casos) correspondió al término previamente acuñado como mioepitelioma, el cual en la clasificación OMS 2022 se designa específicamente como mioepitelioma de las glándulas salivales. El 1% (1 caso) correspondió al término cistadenoma, que en la clasificación actual se considera un término deseable, como cistadenoma de las glándulas salivales. Otro 1% (1 caso) correspondió a una neoplasia benigna que continúa siendo conocida como tumor de Warthin; sin embargo, este caso fue diagnosticado originalmente bajo el término cistadenoma papilar linfomatoso, denominación cuyo uso no se recomienda en la clasificación OMS 2022.

La relevancia de estos cambios radica principalmente en la clarificación y especificación del sitio anatómico de origen, lo cual puede evitar controversias diagnósticas, especialmente en pacientes con antecedentes o presencia de neoplasias en otras glándulas.

Por otro lado, el 2% de los casos (2 casos) correspondió a la eliminación de un término previamente utilizado en la nomenclatura, específicamente el concepto de adenocarcinoma NOS, el cual, en la clasificación OMS 2022, ha sido sustituido por el término carcinoma salival NOS y entidades emergentes. Históricamente, la clasificación de las neoplasias ha buscado encuadrarlas dentro de categorías diagnósticas que aporten información útil para el tratamiento y el pronóstico del paciente. En este contexto, la evolución del término adenocarcinoma NOS hacia el nuevo concepto refleja un esfuerzo por reconocer la heterogeneidad de estas neoplasias y la aparición de entidades emergentes.

Cabe destacar que nuestro estudio carece de los elementos diagnósticos actualmente recomendados para una adecuada caracterización de las neoplasias incluidas en este grupo, lo cual puede atribuirse a la dependencia exclusiva de criterios histopatológicos, sin el apoyo de herramientas diagnósticas complementarias como la inmunohistoquímica o los estudios moleculares, que permiten una clasificación más precisa. Asimismo, el Laboratorio de Patología Oral y Maxilofacial del DEPeI recibe con frecuencia biopsias incisionales, las cuales proporcionan muestras de tejido limitado que pueden no representar de manera completa las características histopatológicas de los tumores de glándulas salivales, al no analizarse la totalidad del espécimen tumoral.

Dentro de los cambios observados, destaca el relacionado con el tumor de Warthin, ya que, aunque esta neoplasia benigna continúa siendo reconocida actualmente bajo dicha denominación, existen diversos términos históricos con los que ha sido designada, lo que ha limitado el consenso en su nomenclatura. Entre los términos no recomendados por la OMS se incluyen adenolinfoma, cistadenoma papilar linfomatoso y cistadenolinfoma. Por lo anterior, se sugiere evitar el uso de estas denominaciones con el fin de prevenir posibles confusiones en el diagnóstico y tratamiento de los pacientes, así como inconsistencias en los registros estadísticos de los tumores de glándulas salivales, al intentar encuadrar esta entidad bajo conceptos distintos al de tumor de Warthin.

Los principales cambios identificados en el presente estudio se relacionaron con la nomenclatura y los conceptos de aquellas entidades que, hasta la actualidad, no han logrado consolidarse como entidades diagnósticas bien definidas. En las clasificaciones de la OMS 2005 y 2017, estas neoplasias se agruparon bajo el término adenocarcinoma NOS, mientras que en la clasificación OMS 2022 se redefinen como carcinoma salival NOS y entidades emergentes, incluso considerando el apoyo de estudios moleculares. Esta situación representa una problemática aún vigente en el ámbito del diagnóstico histopatológico, la cual genera confusión y un bajo grado de consenso entre los patólogos.

Como se observó, los cambios de nomenclatura implicaban añadir o eliminar palabras de nombres previamente establecidos. Por ejemplo, el adenocarcinoma polimorfo de bajo grado (OMS 2005) se denomina ahora adenocarcinoma polimorfo (OMS 2022). Aunque estos cambios pueden causar confusión inicialmente, comprender los criterios detrás de las actualizaciones permite identificar con precisión las entidades.

Este estudio es importante porque ofrece un resumen de estos cambios de nomenclatura y ofrece datos epidemiológicos actualizados de un centro nacional de referencia diagnóstica. También refuerza la necesidad de más investigaciones donde incorporen herramientas diagnósticas tales como la inmunohistoquímica o incluso la secuenciación, así como la consideración de la inclusión de muestras hospitalarias con especímenes quirúrgicos que aporten información completa. Con estos dos parámetros adicionales se podría dar un panorama más específico de los cambios epidemiológicos de tumores de glándulas salivales.

## CONCLUSIONES

Este estudio demuestra que los cambios en la nomenclatura de la OMS para los tumores de glándulas salivales pueden influir de manera significativa en los datos de frecuencia reportados, ya que cada actualización requiere la realización de revisiones diagnósticas que permitan generar información epidemiológica acorde con la clasificación vigente de la OMS.

No obstante, deben señalarse algunas limitaciones del estudio, entre ellas la ausencia de herramientas diagnósticas complementarias, como la inmunohistoquímica y las pruebas moleculares, las cuales proporcionan información más precisa sobre el perfil genotípico de las neoplasias y permiten una mejor asignación a su grupo diagnóstico correspondiente. La incorporación de estas técnicas podría mejorar la precisión diagnóstica y, en consecuencia, ofrecer datos epidemiológicos más confiables.

## Fuentes de financiamiento:

Recursos propios
